# Supplementary material for: RNA binding protein SYNCRIP maintains proteostasis and self-renewal of hematopoietic stem and progenitor cells
Source: Nat Commun. 2023 Apr 21;14:2290. doi: 10.1038/s41467-023-38001-x (PMC10121618; doi:10.1038/s41467-023-38001-x)
Supplement: Supplementary file 1 — Supplementary Information [file 41467_2023_38001_MOESM1_ESM.pdf]

Supplementary Information

RNA binding protein SYNCRIP maintains proteostasis and self-renewal of hematopoietic stem and progenitor cells

Florisela Herrejon Chavez[1,2 #], Hanzhi Luo[1], Paolo Cifani[1,3], Alli Pine[4], Eren L. Chu[1,5], Suhasini Joshi[6], Ersilia Barin[1], Alexandra Schurer[1], Mandy Chan[1], Kathryn Chang[1], Grace YQ Han[1], Aspen J. Pierson[1], Michael Xiao[7], Xuejing Yang[1], Lindsey M Kuehm[8], Yuning Hong[9], Diu T.T. Nguyen[1,10,11], Gabriela Chiosis[6], Alex Kentsis[1,12,13], Christina Leslie[4], Ly P. Vu[1,14,15 #*], Michael G Kharas[1*]

[1] Molecular Pharmacology Program, Memorial Sloan Kettering Cancer Center, New York, NY, USA
[2] Gerstner Sloan Kettering Graduate School of Biomedical Sciences, Memorial Sloan Kettering Cancer Center, New York, NY, USA
[3] Cold Spring Harbor Laboratory, Cold Spring Harbor, NY, USA
[4] Computational Biology Program, Memorial Sloan Kettering Cancer Center, New York, NY, USA
[5] Department of Pharmacology, Weill Cornell School of Medical Sciences, New York, NY, USA
[6] Chemical Biology Program, Memorial Sloan Kettering Cancer Center, New York, NY, USA
[7] Weill Cornell/Rockefeller/Sloan Kettering Tri-Institutional MD-PhD Program, New York, NY, USA
[8] Cell Microsystems, Inc., Durham, NC, USA
[9] Department of Biochemistry and Chemistry, La Trobe University, Melbourne, Australia
[10] Centre for Haemato-Oncology, Barts Cancer Institute, London, UK
[11] Queen Mary University of London, Charterhouse Square, London, UK
[12] Tow Center for Developmental Oncology, Department of Pediatrics, Memorial Sloan Kettering Cancer Center
[13] Departments of Pediatrics, Pharmacology, and Physiology & Biophysics, Weill Medical College of Cornell University.
[14] Terry Fox Laboratory, British Columbia Cancer Research Centre, Vancouver, BC, Canada
[15] Faculty of Pharmaceutical Sciences, University of British Columbia, Vancouver, BC, Canada

[#]These authors contributed equally
*These authors jointly supervised this work: lvu@bccrc.ca and kharasm@mskcc.org

Supplementary Figures
Supplemental Figure 1

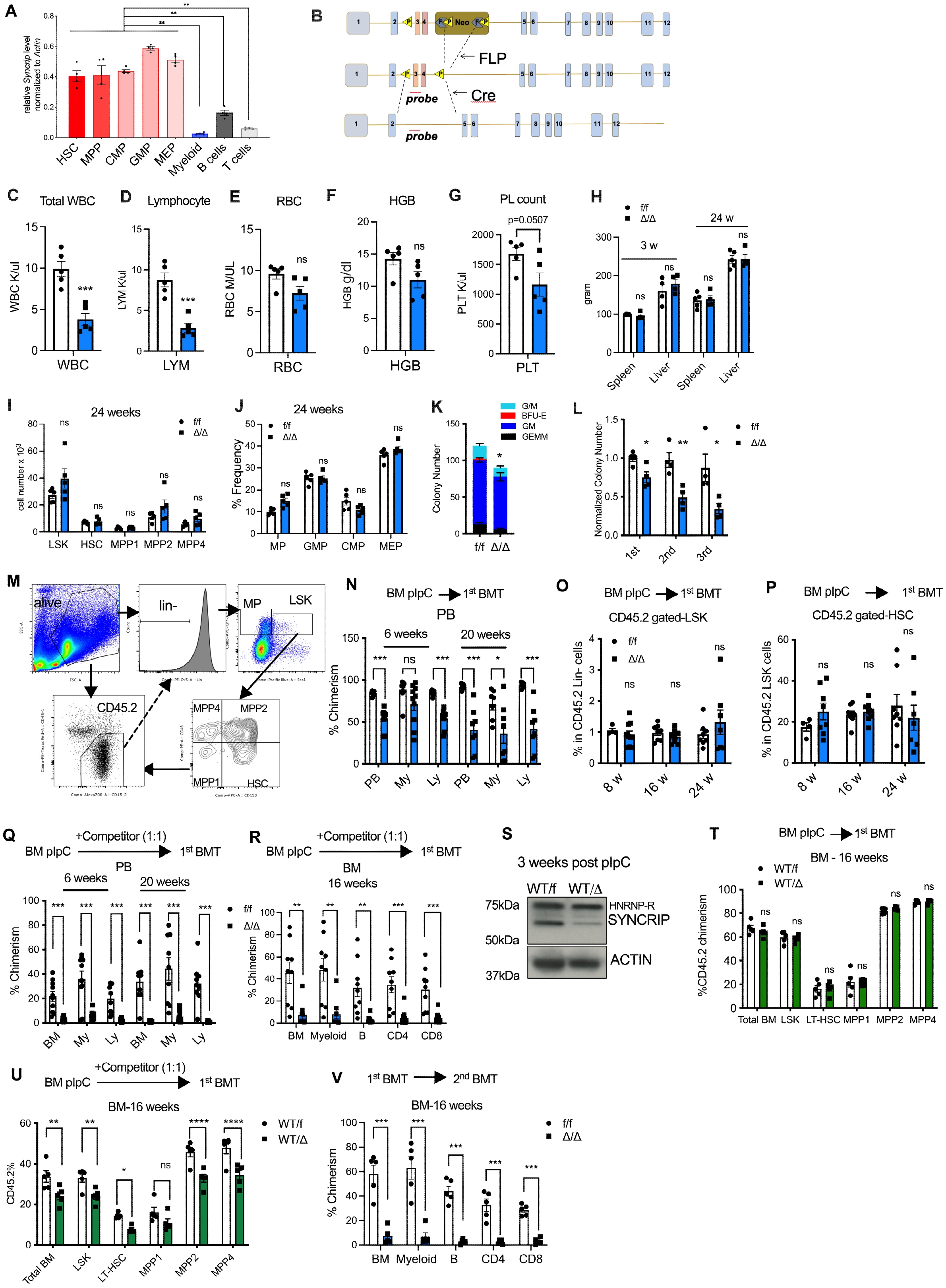

Supplemental Figure 1. SYNCRIP is dispensable for steady state hematopoiesis but required for long term HSC self-renewal

(A) qRT-PCR quantification of *Syncrip* mRNA expression across murine hematopoietic compartments. HSC; MPP; CMP; GMP; MEP; myeloid; B cells and T cells. n=4/cell type, HSC vs Myeloid p=0.0019, HSC vs B-cells p=0.0043, HSC vs T-cell p=0.0032.
(B) Strategy to create *Syncrip* conditional knockout mouse (cKO).
(C-G) Complete blood count (CBC) 16 weeks post pIpC (n=5/phenotype). (C) p=0.0008 (D) p=0.0004 (G) p=0.05
(H) Spleen and liver weight *Syncrip*$^{f/f}$ and *Syncrip*$^{\Delta/\Delta}$ mice 3- and 24 weeks post pIpC (n=4/genotype).
(I) Absolute number of HSC and MPP1, MPP2, and MPP4 (n=5/ genotype).
(J) Frequencies of progenitor populations 24 weeks post pIpC n=5/genotype.
(K) Colony number (n=4/genotype, p=0.0239)
(L) Normalized number of colonies in 1st (p=0.0239), 2nd (p=0.0060), and 3rd (p=0.0288) plating of BM.
(M) Gating strategy for CD45.2 chimerism in stem/progenitor subpopulations.
(N) Chimerism in peripheral blood (PB) (n=2 donor, 6 weeks n=10 recipients/genotype, 20 weeks n=7 recipients/genotype): total PB (p<0.00001/ p=0.0001), My (p=0.0587/ p=0.0260, Ly (p<0.0001/ p<0.0001).
(O-P) Frequencies of (O) LSK and (P) HSC cells within CD45.2+ Lin- and LSK populations, respectively at 8- (*Syncrip*$^{f/f}$ n=4, *Syncrip*$^{\Delta/\Delta}$ n=10), 16- (n=8/genotype) and 24- (n=8 vs. n=7) weeks post transplantation.
(Q-R) Donor chimerism in (Q) PB (p=0.0002/ p <0.0001), My (p=0.0002/ p =0.0003) and Ly (p =0.0001/ p =0.0001_ (n=2 donor, 6 weeks n=10 recipients/genotype, 20 weeks *Syncrip*$^{f/f}$ n=9, *Syncrip*$^{\Delta/\Delta}$ n=10) (R) BM (n=2 donor, and n=10 recipients/genotype) at 16 weeks. BM p=0.0018, Myeloid p =0.0021, B cells p=0.0013, CD4 p =0.0006, CD8 p=0.0007.
(S) Immunoblots showing SYNCRIP depletion in BM of heterozygous knockout *Syncrip* $^{f/\Delta}$ mice. ACTIN as loading control.
(T) Donor chimerism at 16 weeks post-transplantation.
(U) Donor chimerism of competitive transplant of *Syncrip*$^{f/f}$ and *Syncrip*$^{f/\Delta}$ 16 weeks post-transplantation. (n=5 recipients/genotype). Total BM p=0.0019. LSK p=0.0031, HSC p=0.0236, MPP2 p<0.0001, MPP4 p<0.0001.
(V) Donor chimerism in BM of secondary recipients at 16 weeks post-transplantation.(n=2 donor, and n=10 recipients/genotype). p-value<0.001 for all parameters.
Source data are provided as a Source Data file. All data represent mean ± s.e.m. p values were calculated by two-tailed t test unless specified. * p<0.05, **p<0.01, ***p<0.001 and ns: not significant.

Supplemental Figure 2

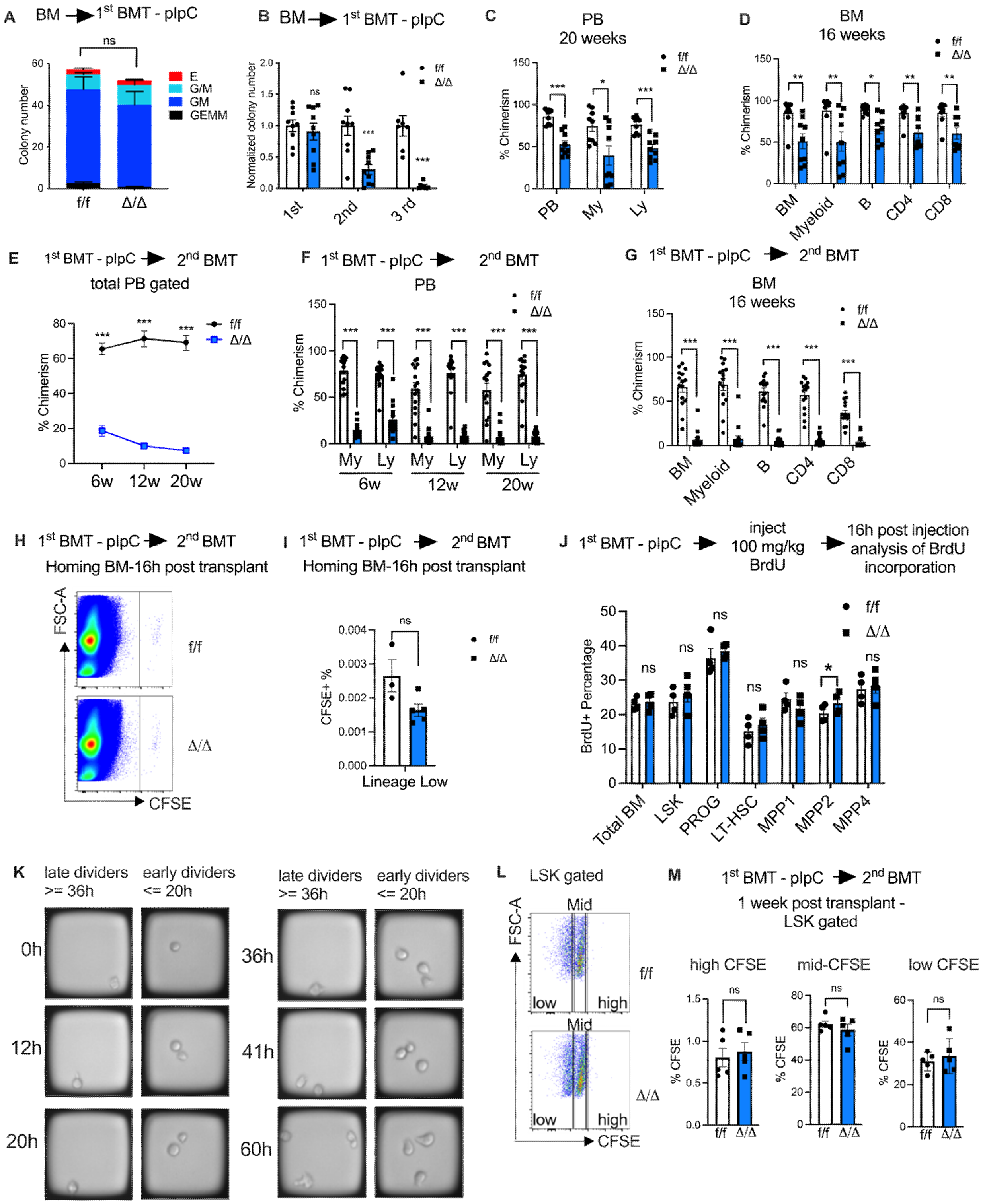

Supplemental Figure 2. SYNCRIP plays a critical role in stress hematopoiesis

(A) The number of colonies formed from *Syncrip*$^{f/f}$ and *Syncrip*$^{\Delta/\Delta}$ BM cells (described in Figure 2A). (n=9/genotype).
(B) Normalized number of colonies formed in sequential 1st, 2nd and 3rd plating of cells described in (A). 2nd and 3rd plating p<0.0001.
(C) Donor chimerism in PB 20- weeks post transplantation (n=3 donor, and n=9 recipients/genotype). Total PB value<0.0001, My p=0.0175, Ly p=0.0001
(D) Donor chimerism in BM 16 weeks post transplantation (n=3 donor, and n=9 recipients/genotype). BM p=0.0046, Myeloid p=0.0133, B p=0.0007, CD4 p=0.0039, CD8 p=0.0064.
(E) Donor chimerism in PB of secondary recipients (described in Figure 2E) at 6-, 12- and 20 weeks post transplantation (n=3 donor, and n=15 recipients/genotype). p-value<0.0001 for all timepoints.
(F) Donor chimerism in PB populations of secondary recipients described in (F) at 6-, 12- and 20 weeks post transplantation (n=3 donor, and n=15 recipients/genotype). p-value<0.0001 for all conditions.
(G) Donor chimerism within BM populations in secondary recipients described in (E) at 16 weeks post-transplantation (n=3 donor, and n=15 recipients/genotype). p-value<0.0001 for all conditions.
(H) Representative flow plots showing gating of CFSE positive LSK cells engrafted in BM of secondary recipients at 16 hours post-transplantation (n=5/genotype).
(I) Quantitative summary of data shown in (H) *Syncrip*$^{f/f}$ (n=3) and *Syncrip*$^{\Delta/\Delta}$ (n=5)
(J) Incorporation of BrdU pulse into stem/progenitor compartments of *Syncrip*$^{f/f}$ and *Syncrip*$^{\Delta/\Delta}$. n=4/genotype. MPP2 p=0.0134.
(K) Representative images of *in vitro* HSC cell division (described in Figure 2K) imaged on CellRaft AIR System (Cell Microsystems), shown in brightfield.
(L)Representative flow plots showing gating strategy of CFSE stained LSK in recipient mice (described in E) at 1 week post transplantation.
(M) Quantitative summary of data shown in (J). n=5/genotype
Source data are provided as a Source Data file. All data represent mean ± s.e.m. p values were calculated by two-tailed t test unless specified. * p<0.05, **p<0.01, ***p<0.001 and ns: not significant.

Supplemental Figure 3

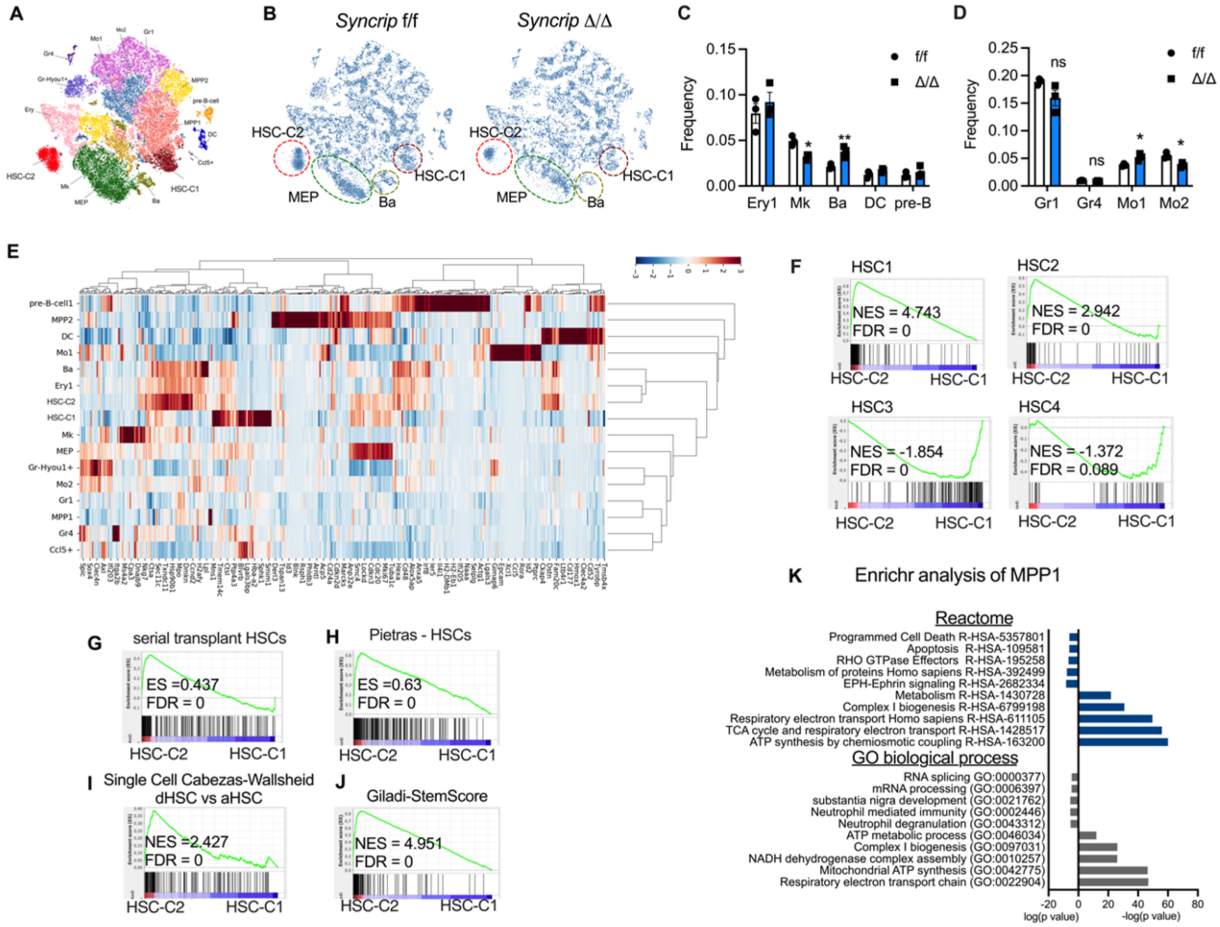

Supplemental Figure 3. Single cell RNA sequencing (scRNA-seq) uncovered an activated unfolded protein response in *Syncrip* deficient HSC populations

(A) Identification of hematopoietic cell populations within WT *Syncrip*$^{f/f}$ (n=3) and KO *Syncrip*$^{\Delta/\Delta}$ (n=3) Lin-ckit+ cells based on tSNE analysis of Single cell RNA sequencing (scRNA-seq).
(B) tSNE displays of all hematopoietic clusters of WT *Syncrip*$^{f/f}$ and KO *Syncrip*$^{\Delta/\Delta}$ scRNA-seq as described in (Figure 3E). Cluster HSC-C2 shows the most shift. HSC-C1, Ba and MEP clusters were highlighted for comparison.
(C-D) Quantitative summary of frequencies of different populations defined by scRNA-seq analysis in WT *Syncrip* f/f (n=3) and KO *Syncrip* Δ/Δ (n=3). Data represent mean $\pm$ s.e.m. p values were calculated by two-tailed t test unless specified. * $p<0.05$, ** $p<0.01$, ns: not significant. Mk p=0.009, Ba p=0.0132, Mo1 p=0.0235, Mo2 p=0.0139.
(E) Heat map showing relative gene expression across all cell types of all marker genes used in Louvain clusters to generate hematopoietic clusters depicted in Figure 3 and Supplemental figure 3.
(F) GSEA analysis of HSC-C2 (vs HSC-C1) transcriptome against those of different HSC clusters defined in Rodriguez-Fraticelli et al., 2020.
(G-J) GSEA analysis of HSC-C2 (vs. HSC-C1) transcriptome against signatures of (G) serial transplant HSCs, (H) primitive HSCs, (I) dormant HSCs and (J) StemScore.
(K) Enrichr analysis for GO biological processes and Reactome enrichment of significant (FDR <0.05) downregulated and upregulated genes within MPP1 populations of *Syncrip*$^{\Delta/\Delta}$ vs. *Syncrip*$^{f/f}$ based on sc-RNA seq analysis. X-axis: $-\log_{10}$(p value). *p*-values were calculated by Fisher's exact test. Source data are provided as a Source Data file.

Supplemental Figure 4

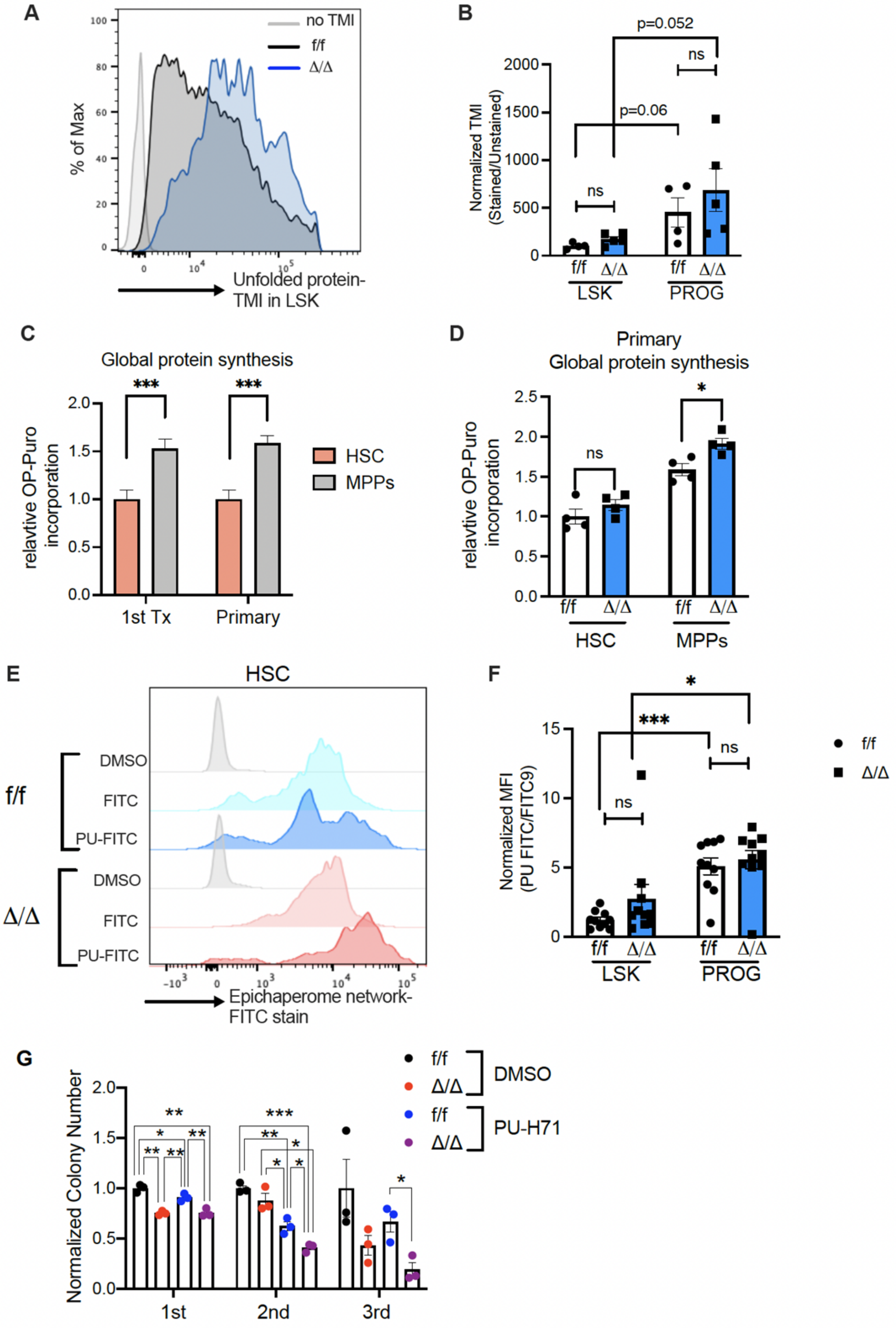

Supplemental Figure 4. Deletion of SYNCRIP deregulates the proteostasis network in HSCs

(A) Representative histograms of TMI flow analysis of LSK (Lin-Sca+ckit+) cells from *Syncrip*$^{f/f}$ vs. *Syncrip*$^{\Delta/\Delta}$ mice.
(B) Quantitative summary of relative tetraphenylethene maleimide (TMI) fluorescent signals in LSK and PROG (from *Syncrip*$^{f/f}$ (n=4) vs. *Syncrip*$^{\Delta/\Delta}$ (n=5). LSK vs PROG (Lin-Sca-ckit+). *Syncrip*$^{f/f}$ p=0.06, *Syncrip*$^{\Delta/\Delta}$ p=0.052
(C) OP-Puro incorporation in hematopoietic stem cell (HSC) and multipotent-progenitor (MPP) populations isolated from *Syncrip* f/f primary mice (n=4) (described in Figure 1) or *Syncrip* f/f 1st transplant recipient mice (n=5) (described in Figure 2). OP-Puro incorporation was used to quantify the level of global protein synthesis in single hematopoietic cells. p<0.01 for all comparisons.
(D) OP-Puro incorporation in HSC (CD150+ CD48- LSK) and MPP (CD150- and CD150+CD48+ LSK) populations isolated from *Syncrip*$^{f/f}$ (n=5) vs. *Syncrip*$^{\Delta/\Delta}$ (n=5) primary mice. OP-Puro incorporation was used to quantify the level of global protein synthesis in single hematopoietic cells. MPPs p=0.0167.
(E) Representative histograms of PU-FITC flow analysis of HSCs from *Syncrip* $^{f/f}$ vs. *Syncrip* $^{\Delta/\Delta}$. DMSO – stain control; FITC – background signal control and PU-FITC corresponding to level of epichaperome in single hematopoietic cells.
(F) Quantitative summary of PU-FITC fluorescent signals normalized to FITC control in LSK and PROG. PU-FITC signals were used to quantify the epichaperome single hematopoietic cells in *Syncrip* $^{f/f}$ (n=10) vs. *Syncrip* $^{\Delta/\Delta}$ (n=10). LSK vs PROG. *Syncrip*$^{f/f}$ p<0.0001, *Syncrip*$^{\Delta/\Delta}$ p=0.0338.
(G) Normalized number of colonies formed in sequential 1st, 2nd and 3rd plating of LSK cells isolated from *Syncrip*$^{f/f}$ and *Syncrip*$^{\Delta/\Delta}$ mice and treated with either vehicle- control or Epichaperome inhibitor PU-H17 (n=3/each condition). DMSO *Syncrip*$^{f/f}$ vs *Syncrip*$^{\Delta/\Delta}$ 1st plating p=0.0006, *Syncrip*$^{f/f}$ DMSO vs PU-H71 1st plating p=0.0406, 2nd plating p=0.002. DMSO *Syncrip*$^{f/f}$ vs PU-H71 *Syncrip*$^{\Delta/\Delta}$ 1st plating p=0.0016, 2nd plating p<0.0001. DMSO *Syncrip*$^{\Delta/\Delta}$ vs PU-H71 *Syncrip*$^{f/f}$ 1st plating p=0.0034, 2nd plating p=0.035. DMSO *Syncrip*$^{\Delta/\Delta}$ vs PU-H71 *Syncrip*$^{f/f}$ 2nd plating p=0.0030. PU-H71 *Syncrip*$^{f/f}$ vs PU-H71 *Syncrip*$^{\Delta/\Delta}$ 1st plating p=0.0091, 2nd plating p=0.0147, 3rd plating p=0.0192.
Source data are provided as a Source Data file. All data represent mean ± s.e.m. p values were calculated by two-tailed t test unless specified. * p<0.05, **p<0.01, ***p<0.001 and ns: not significant.

Supplemental Figure 5

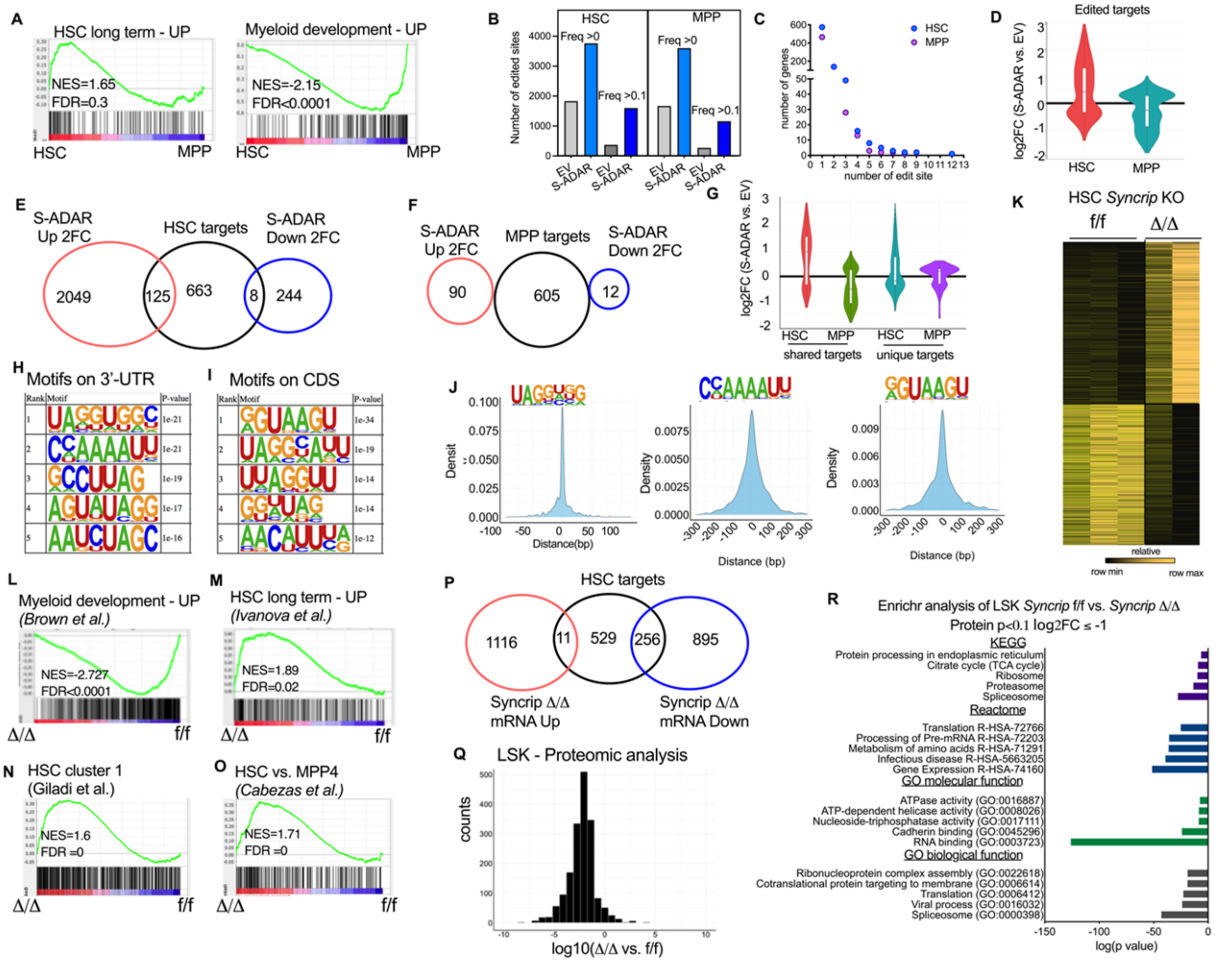

Supplemental Figure 5. Multi-omics analysis identifies SYNCRIP functional targets in HSCs

(A) GSEA analysis of genes differentially regulated in hematopoietic stem cells (HSCs) vs. multipotent-progenitor (MPP) cells (described in Figure 5A).
(B) Number of total ADAR-mediated edit sites with Frequency (Freq) >0 and Freq >0.1 in HSC and MPP.
(C) Distribution of number of genes with corresponding number of edit sites.
(D) Violin plots showing mRNA expression (FDR <0.05, log2FC S-ADAR vs. EV) of SYNCRIP direct targets identified in HSC (n=796) and MPP (n=605). Box plots and whiskers representing minimum value (lowest bound); maximum value (highest bound); a range of lower quartile (the start of the box) and the upper quartile (the end of the box) and median as the middle line.
(E) Venn diagram showing SYNCRIP targets in HSCs with genes differentially upregulated (n=2174) and downregulated (n=252) in HSCs.
(F) Venn diagram showing SYNCRIP targets in MPP with genes differentially upregulated (n=90) and downregulated (n=12) in MPP.
(G) Violin plots showing level of mRNA expression (FDR <0.05, log2FC S-ADAR vs. EV) of shared targets (n=534), HSC unique targets (n=262) and MPP (n=71) unique targets defined in Figure 5C.
(H-I) SYNCRIP specific binding motifs enriched in coding regions (CDS) and (I) 3'-UTR.
(J) Probability density function (PDF) plots showing the distance from edits sites to the nearest SYNCRIP motifs.
(K) Heatmap of the top upregulated and downregulated genes (fold change ≥ 2, FDR <0.05) from RNA sequencing analysis of HSCs *Syncrip*$^{f/f}$ (n=3) and *Syncrip*$^{\Delta/\Delta}$ (n=2) described in Figure 2.
(L-O) GSEA analysis of targets differentially regulated in HSCs of *Syncrip*$^{\Delta/\Delta}$ vs. *Syncrip*$^{f/f}$ for signatures pertaining to (L) Myeloid development, (M-O) long term HSCs.
(P) Venn diagram showing SYNCRIP target mRNAs in HSCs with genes whose expression levels are altered (FDR <0.05, FC≥0) in *Syncrip* deficient HSCs.
(Q) Absolute counts representing the abundance of proteins distributed across the range of $\log_{10}$foldchange in LSK cells (n=5 replicates of total 15 mice/genotype).
(R) Enrichr analysis for GO biological processes, GO molecular function, Reactome and KEGG enrichment of SYNCRIP target genes whose proteins are downregulated in *Syncrip* deficient LSKs. X-axis: $\log_{10}$(p value). *p*-values were calculated by Fisher's exact test.

Supplemental Figure 6

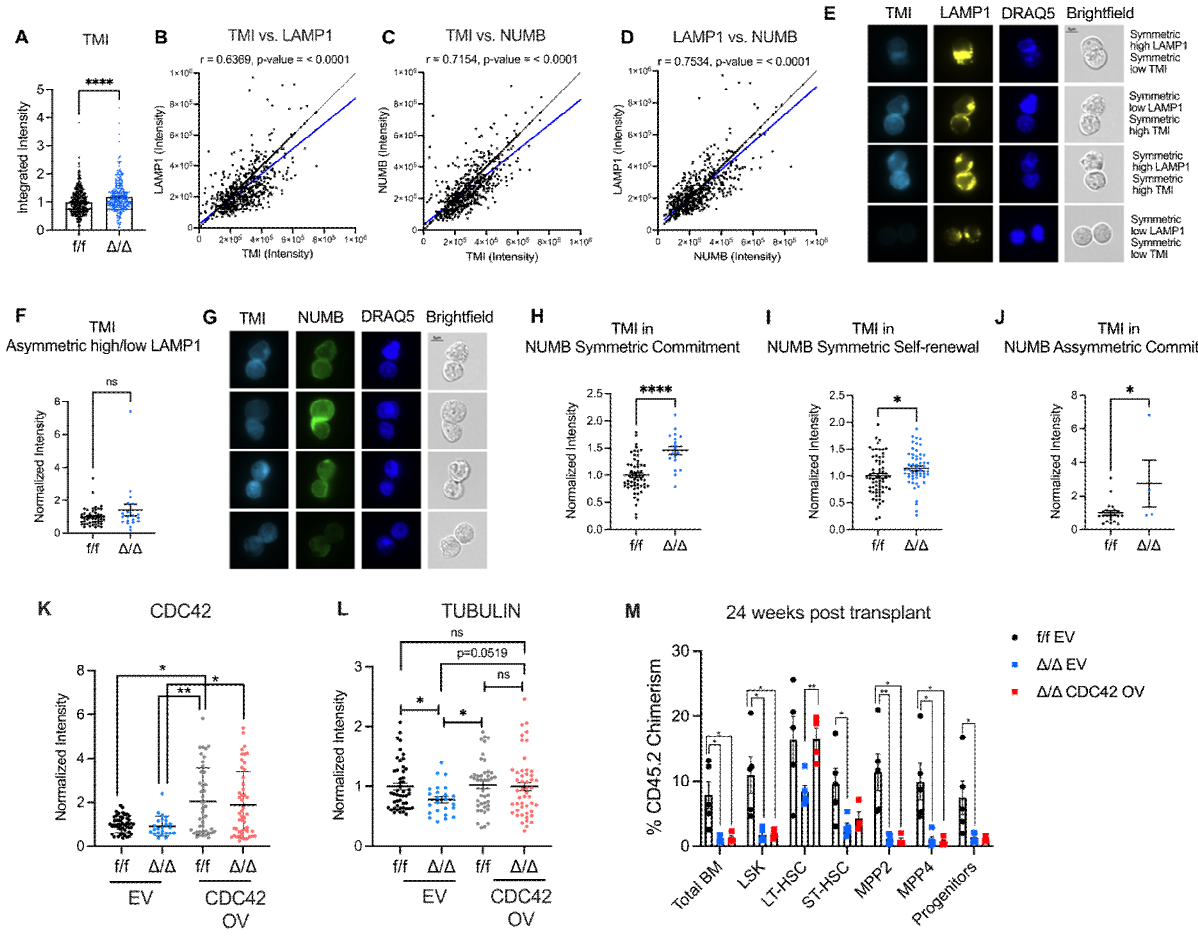

Supplemental Figure 6. SYNCRIP controls HSC polarity and symmetric vs. asymmetric division

(A) Quantification of normalized TMI IF intensity in HSCs *Syncrip*$^{f/f}$ n=565 and *Syncrip*$^{\Delta/\Delta}$ n=378.
(B-C) Correlation between TMI signals and (B) LAMP1 and (C) NUMB protein levels. *p*-values were calculated by Pearson r correlation test. p<0.0001 for all correlation tests.
(D) Correlation between LAMP1 and NUMB protein levels. *p*-values were calculated by Pearson r correlation test. p<0.0001 for correlation test.
(E) Representative images of IF staining of TMI and LAMP1 in paired daughter assay performed with *Syncrip*$^{f/f}$ and *Syncrip*$^{\Delta/\Delta}$ HSCs. TMI (left); LAMP1 (middle), DRAQ5 (right) and brightfield.
(F) Quantification of normalized IF intensity reflecting the levels of TMI in doublet cells with asymmetric high/low LAMP1 level in *Syncrip*$^{f/f}$ n=46 and *Syncrip*$^{\Delta/\Delta}$ n=20.
(G) Representative images of immunofluorescence (IF) staining of TMI and NUMB immunofluorescence (IF) staining in paired daughter assay performed with *Syncrip*$^{f/f}$ and *Syncrip*$^{\Delta/\Delta}$ HSCs. TMI (left); NUMB (middle), DRAQ5 (right) and brightfield.
(H-J) Quantification of normalized TMI IF intensity in doublet cells of NUMB paired daughter assay (H) NUMB symmetric commitment *Syncrip*$^{f/f}$ n=57 and *Syncrip*$^{\Delta/\Delta}$ n=18.
(I) NUMB symmetric self-renewal *Syncrip*$^{f/f}$ n=64 and *Syncrip*$^{\Delta/\Delta}$ n=54 and (J) NUMB asymmetric commitment *Syncrip*$^{f/f}$ n=22 and *Syncrip*$^{\Delta/\Delta}$ n=4.
(K-L) Quantification of normalized IF intensity reflecting the protein levels of (K) CDC42 and (L) TUBULIN in *Syncrip*$^{f/f}$ and *Syncrip*$^{\Delta/\Delta}$ LSK cells transduced with either empty vector control (EV) or vector expressing CDC42 (CDC42-OV) as described in Figure 6S-T. EV *Syncrip*$^{f/f}$ n=48; EV *Syncrip*$^{\Delta/\Delta}$ n=24; CDC42-OV *Syncrip*$^{f/f}$ n=42; CDC42-OV *Syncrip*$^{\Delta/\Delta}$ n=49.
(M) Donor chimerism of LSK cells isolated from *Syncrip*$^{f/f}$ and *Syncrip*$^{\Delta/\Delta}$ mice and transduced with either empty vector control (EV) or vector expressing CDC42 (CDC42-OV) as described in figure 6S in primary recipients (n=5 mice/each condition).
All scale bars 5μm. All data represent mean ± s.e.m. p values were calculated by two-tailed t test unless specified. * p<0.05, **p<0.01, ***p<0.001 and ns: not significant. Source data are provided as a Source Data file.

Supplementary File Western Blots Uncropped:

Supplementary Figure 1S

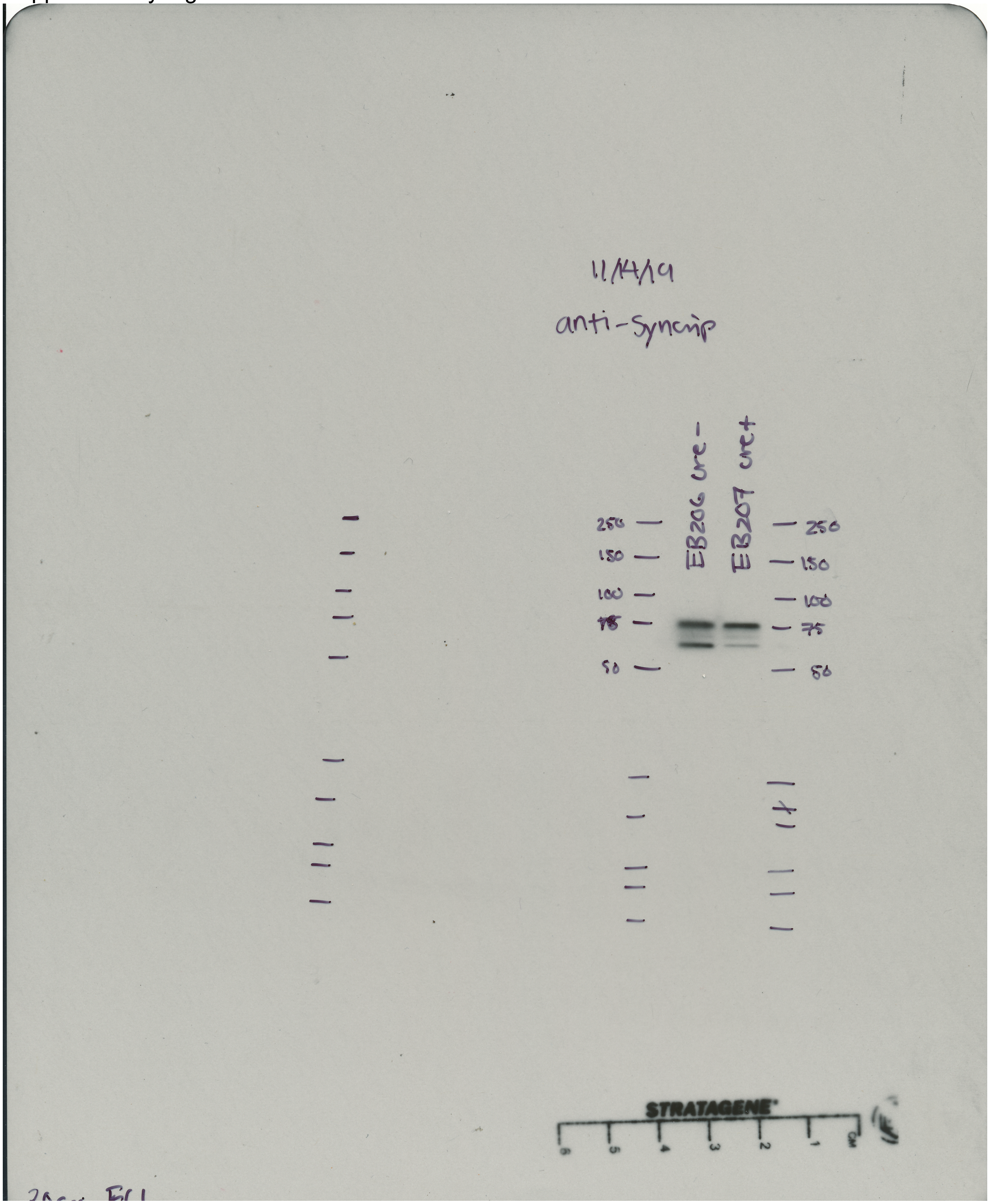

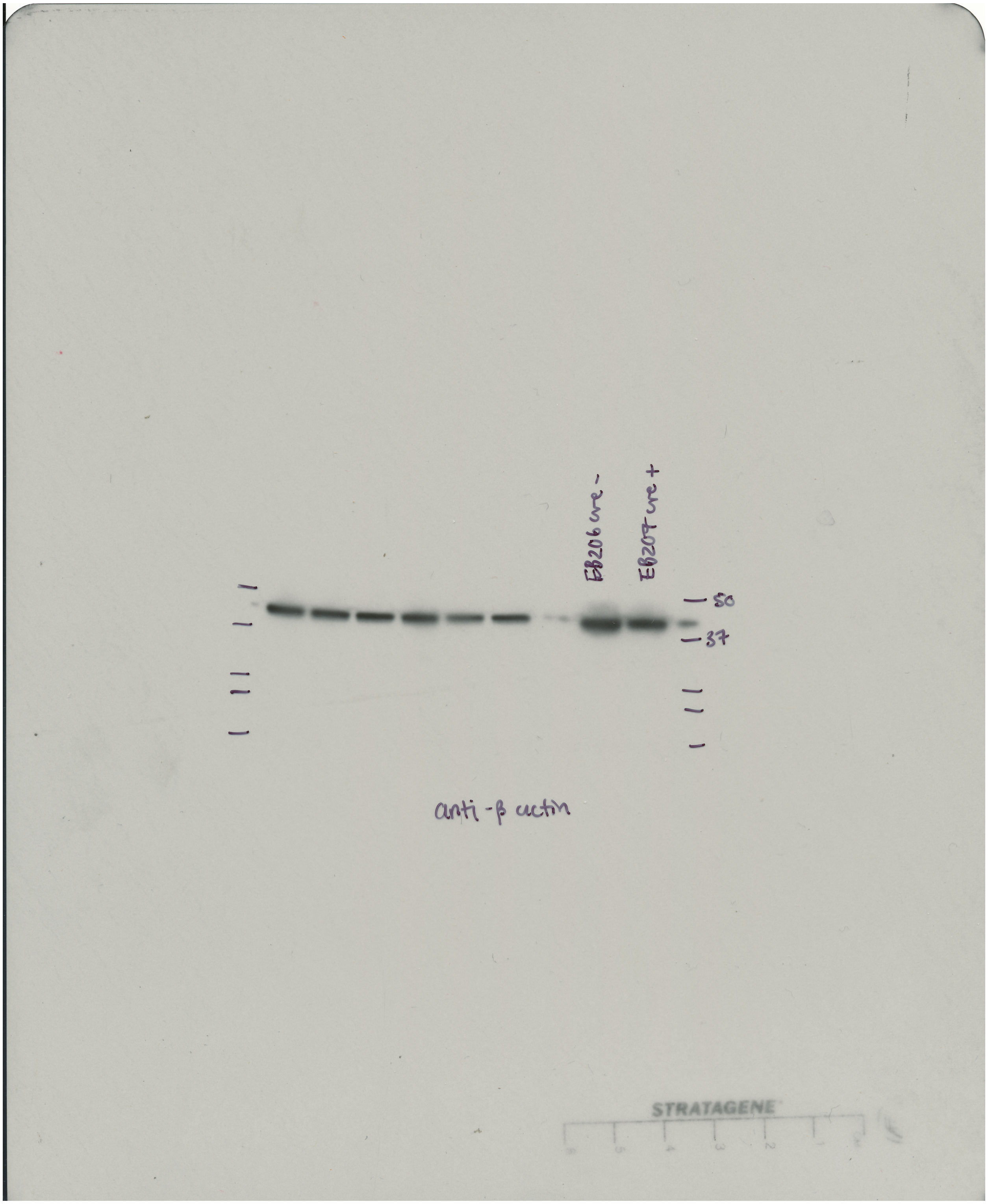
EB206 cre -
EB207 cre +
50
37
anti-β actin
STRATAGENE
