# Supplementary material for: RNA binding protein SYNCRIP maintains proteostasis and self-renewal of hematopoietic stem and progenitor cells
Source: Nat Commun. 2023 Apr 21;14:2290. doi: 10.1038/s41467-023-38001-x (PMC10121618; doi:10.1038/s41467-023-38001-x)
Supplement: Supplementary file 3 — Description of Additional Supplementary Files [file 41467_2023_38001_MOESM3_ESM.pdf]

Description of Additional Supplementary Files

File Name: Supplementary Data 1
Description: scRNA-seq sample details. Tables contains information of samples used for scRNA-sequencing including number of cells and genes for each replicate of both genotypes *Syncrip* f/f (n=3) and *Syncrip* Δ/Δ (n=3).

File Name: Supplementary Data 2
Description: scRNA-seq cluster vs rest DEG. Differentially expressed genes between a cluster vs. the rest of other clusters characterized in scRNA-seq datasets. Total 21 clusters as defined in cluster assignment. The statistical test was performed using DEseq Bioconductor package. In general, DEseq algorithm performs estimation of size factors and estimation of dispersion prior to negative binomial GLM fitting and Wald statistic. A one-sided Wilcoxon test was performed to determine the statistical significance between the log2 fold changes (log2FC).

File Name: Supplementary Data 3
Description: scRNA-seq HSC C1 vs HSC C2 pval rank list. Table showing ranked list of all differentially expressed genes between WT HSC C1 vs HSC C2 from scRNA-seq results. Table was used as a rnk file to run GSEA with other publicly available datasets.

File Name: Supplementary Data 4
Description: scRNA-seq frequency of clusters. Table showing frequencies of clusters defined in scRNA-seq datasets. Frequencies were shown for each individual replicate in both genotypes *Syncrip* f/f (n=3) and *Syncrip* Δ/Δ (n=3). Student t-test two sided.

File Name: Supplementary Data 5
Description: scRNA-seq WT vs KO all clusters. Table showing differential gene expression analysis between *Syncrip* f/f (n=3) and *Syncrip* Δ/Δ (n=3) of all matched clusters characterized in scRNA-seq datasets. The statistical test was performed using DEseq Bioconductor package. In general, DEseq algorithm performs estimation of size factors and estimation of dispersion prior to negative binomial GLM fitting and Wald statistic. A one-sided Wilcoxon test was performed to determine the statistical significance between the log2 fold changes (log2FC).

File Name: Supplementary Data 6
Description: Enrichr analysis of HSC C2 KO vs WT. Table showing results of gene set enrichment analysis using https://maayanlab.cloud/Enrichr/ of differentially expressed genes (FDR <=0.05; up and down regulated genes) within cluster HSC C2 between *Syncrip* f/f (n=3) and *Syncrip* Δ/Δ (n=3). Pathway analysis on GO biological process 2021 and Reactome 2016. The analysis and statistical test were performed using https://maayanlab.cloud/Enrichr/.
In brief, Enrichr analysis includes three enrichment scores based on three statistical tests: 1) the Fisher exact test; 2) the z-score calculating deviation from the expected rank by the Fisher exact test; and 3) a combined score which is the multiplication of the log p value obtained from the test 1 and test 2.

File Name: Supplementary Data 7
Description: Enrichr analysis of HSC C1 KO vs WT. Table showing results of gene set enrichment analysis using https://maayanlab.cloud/Enrichr/ of differentially expressed genes (FDR <=0.05; up and down regulated genes) within cluster HSC C1 between *Syncrip* f/f (n=3) and *Syncrip* Δ/Δ (n=3). Pathway analysis on GO biological process 2021 and Reactome 2016. The analysis and statistical test were performed using https://maayanlab.cloud/Enrichr/. In brief, Enrichr analysis includes three enrichment scores based on three statistical tests: 1) the Fisher exact test; 2) the z-score calculating deviation from the expected rank by the Fisher exact test; and 3) a combined score which is the multiplication of the log p value obtained from the test 1 and test 2.

File Name: Supplementary Data 8
Description: HSC vs MPP DEG. Table showing gene IDs and RPKM (reads per kilobases of transcript per 1 million mapped reads) of all genes measured in HSCs vs. MPPs in RNA-sequencing datasets when cells were transduced with empty vector.

File Name: Supplementary Data 9
Description: Syncrip HyperTRIBE significant edited sites HSCs and MPPs. Table showing significant edited sites identified with HyperTRIBE in HSCs and MPPs (fpkm > 5, p-adj > 0.05, differential edit frequency > 0.1) To define difference in editing frequencies, we used beta-binomidal distribution and p values were were adjusted to control for false discovery rate (FDR) using a Benjamin–Hochberg correction. The statistical computation was performed using R packages VGAM (Version 1.1–2) and bbmle (Version 1.0.23.1) as described in Nguyen DTT, et al. 2020

File Name: Supplementary Data 10
Description: SYNCRIP direct targets in HSCs and MPPs. Table showing gene IDs of SYNCRIP's direct transcript targets in HSCs and MPPs and the shared targets.

File Name: Supplementary Data 11
Description: Enrichr analysis of SYNCRIP shared targets in HSCs and MPPs. Table showing results of gene set enrichment analysis using https://maayanlab.cloud/Enrichr/ of shared direct targets of SYNCRIP identified in both HSC and MPP subpopulations. Pathway analysis on GO biological process 2021, GO molecular function 2021, Reactome 2016, KEGG 2021

File Name: Supplementary Data 12
Description: HSC RNA-seq KO vs WT DEG. Table showing gene IDs and RPKM (reads per kilobases of transcript per 1 million mapped reads) of all genes measured in bulk RNA-seq of HSCs of both genotypes *Syncrip* f/f (n=3) and *Syncrip* Δ/Δ (n=3). The statistical test was performed using DEseq Bioconductor package. In general, DEseq algorithm performs estimation of size factors and estimation of dispersion prior to negative binomial GLM fitting and Wald statistic. A one-sided Wilcoxon test was performed to determine the statistical significance between the log2 fold changes (log2FC).

File Name: Supplementary Data 13
Description: HSC RNA-seq KO vs WT DEG rank list. Table showing ranked list used for GSEA analysis of differential gene expression in HSCs of *Syncrip* f/f (n=3) and *Syncrip* Δ/Δ (n=3).

File Name: Supplementary Data 14
Description: Enrichr analysis on HSC RNA-seq KO vs WT. Table showing results of gene set enrichment analysis using https://maayanlab.cloud/Enrichr/ of differentially expressed genes (FDR <=0.05; up and down regulated genes) in buld RNA-seq of HSCs between *Syncrip* f/f (n=3) and *Syncrip* Δ/Δ (n=3). Pathway analysis on GO biological process 2021 and Reactome 2016.

File Name: Supplementary Data 15
Description: Mass spectrometry analysis of LSKs KO vs WT. Table showing protein IDs and proteomic changes in LSK cells of *Syncrip* f/f (n=3) and *Syncrip* Δ/Δ (n=3). A one-sided Wilcoxon test was performed to determine the statistical significance between the log2 fold changes (log2FC).

File Name: Supplementary Data 16
Description: Overlapping SYNCRIP targets. Table showing gene IDs of hits definied as overlapped genes identified from various omic approaches (hyperTribe, RNA-seq, and mass spectrometry).

File Name: Supplementary Data 17
Description: Enrichr analysis on SYNCRIP overlapped targets. Table showing results of gene set enrichment analysis using https://maayanlab.cloud/Enrichr/ of overlapped SYNCRIP direct targets. Pathway analysis on GO biological process 2021, GO molecular function 2021, Reactome, 2016, and KEGG 2021. For directionality, proteomics results were used (p-value <0.1, Log2FC ≤ -1). The analysis and statistical test were performed by https://maayanlab.cloud/Enrichr/. In brief, Enrichr analysis includes three enrichment scores based on three statistical tests: 1) the Fisher exact test; 2) the z-score calculating deviation from the expected rank by the Fisher exact test; and 3) a combined score which is the multiplication of the log p value obtained from

File Name: Supplementary Movie 1
Description: Representative videos for CDC42 Rescue in LSK cells described in Figure 6S-T. Videos show GFP expression in green, tubulin in red, CDC42 in magenta and DAPI counterstain in blue. Only cells with GFP integrated intensities > 100000 were used for quantification in Figure 6T. Movie 1 represents *Syncrip*$^{f/f}$ LSK cells that were transduced with empty vector (EV) MIGR1 retrovirus.

File Name: Supplementary Movie 2
Description: Representative videos for CDC42 Rescue in LSK cells described in Figure 6S-T. Videos show GFP expression in green, tubulin in red, CDC42 in magenta and DAPI counterstain in blue. Only cells with GFP integrated intensities > 100000 were used for quantification in Figure 6T. Movie 2 represents *Syncrip*$^{\Delta/\Delta}$ LSK cells that were transduced with empty vector (EV) MIGR1 retrovirus.

File Name: Supplementary Movie 3
Description: Representative videos for CDC42 Rescue in LSK cells described in Figure 6S-T. Videos show GFP expression in green, tubulin in red, CDC42 in magenta and DAPI counterstain in blue. Only cells with GFP integrated intensities > 100000 were used for quantification in Figure 6T. Movie 3 represents *Syncrip*$^{\Delta/\Delta}$ LSK that were transduced with CDC42 Overexpression (OV) retrovirus.

File Name: Supplementary Movie 4
Description: Representative videos for CDC42 Rescue in LSK cells described in Figure 6S-T. Videos show GFP expression in green, tubulin in red, CDC42 in magenta and DAPI counterstain in blue. Only cells with GFP integrated intensities > 100000 were used for quantification in Figure 6T. Movie 4 represents *Syncrip*$^{f/f}$ LSKs that were transduced with CDC42 Overexpression (OV) retrovirus.
