# Supplementary material for: RNA binding protein SYNCRIP maintains proteostasis and self-renewal of hematopoietic stem and progenitor cells
Source: Nat Commun. 2023 Apr 21;14:2290. doi: 10.1038/s41467-023-38001-x (PMC10121618; doi:10.1038/s41467-023-38001-x)
Supplement: Supplementary file 25 — Reporting Summary [file 41467_2023_38001_MOESM25_ESM.pdf]

nature portfolio

Corresponding author(s): Michael Kharas, Ly Vu

Last updated by author(s): Apr 5, 2023

Reporting Summary

Nature Portfolio wishes to improve the reproducibility of the work that we publish. This form provides structure for consistency and transparency in reporting. For further information on Nature Portfolio policies, see our Editorial Policies and the Editorial Policy Checklist.

Statistics

For all statistical analyses, confirm that the following items are present in the figure legend, table legend, main text, or Methods section.

n/a	Confirmed	
☐	☒	The exact sample size (*n*) for each experimental group/condition, given as a discrete number and unit of measurement
☐	☒	A statement on whether measurements were taken from distinct samples or whether the same sample was measured repeatedly
☐	☒	The statistical test(s) used AND whether they are one- or two-sided<br>*Only common tests should be described solely by name; describe more complex techniques in the Methods section.*
☒	☐	A description of all covariates tested
☒	☐	A description of any assumptions or corrections, such as tests of normality and adjustment for multiple comparisons
☐	☒	A full description of the statistical parameters including central tendency (e.g. means) or other basic estimates (e.g. regression coefficient) AND variation (e.g. standard deviation) or associated estimates of uncertainty (e.g. confidence intervals)
☐	☒	For null hypothesis testing, the test statistic (e.g. *F*, *t*, *r*) with confidence intervals, effect sizes, degrees of freedom and *P* value noted<br>*Give P values as exact values whenever suitable.*
☒	☐	For Bayesian analysis, information on the choice of priors and Markov chain Monte Carlo settings
☒	☐	For hierarchical and complex designs, identification of the appropriate level for tests and full reporting of outcomes
☒	☐	Estimates of effect sizes (e.g. Cohen's *d*, Pearson's *r*), indicating how they were calculated

Our web collection on statistics for biologists contains articles on many of the points above.

Software and code

Policy information about availability of computer code

Data collection	All software used for data collection is commercially available. Those include: Zeiss Zen microscopy software. For flow cyotometry and FACS data collection BD Fortessa or Aria equipment with BD FACSDiva software v1.0 were used. For RNA-seq data collection, Illumina HiSeq platform was used. Complete blood counts were collected on Heska Element HT5 instrument, using HeskaView Integrated Software Version 3.2.3.
Data analysis	Statistical analysis was performed using Prism Graphpad (https://www.graphpad.com/scientific-software/prism/). Differential expression in RNA-seq data in HSC SYNCRIP f/f vs. SYNCRIP KO cells was analyzed using DESeq2 R package. Hypertribe analysis was performed using Hypertribe pipeline previously reported https://github.com/DiuTTNguyen/MSI2_HyperTRIBE_codes. Pathway analysis was performed using Gene Set Enrichment Analysis (GSEA) (https://www.gsea-msigdb.org/gsea/index.jsp) and Enrichr (https://maayanlab.cloud/Enrichr/). Single-cell RNA-seq data was processed and aligned to mouse reference mm10 using CellRanger (10Xgenomics) with default parameters. Normalization, dimensionality reduction and Louvain clustering were performed using Scanpy (v1.4.4 https://scanpy.readthedocs.io/en/stable/). For microscopy image, analysis ImageJ Version 2.0.0-rc-65/1.51w was used. Flow Cytometry data was analyzed on FlowJo version 10.8.1.

For manuscripts utilizing custom algorithms or software that are central to the research but not yet described in published literature, software must be made available to editors and reviewers. We strongly encourage code deposition in a community repository (e.g. GitHub). See the Nature Portfolio guidelines for submitting code & software for further information.

Data

Policy information about availability of data

All manuscripts must include a data availability statement. This statement should provide the following information, where applicable:

- Accession codes, unique identifiers, or web links for publicly available datasets
- A description of any restrictions on data availability
- For clinical datasets or third party data, please ensure that the statement adheres to our policy

RNA-seq that support the findings in this study have been deposited in the Gene Expression Omnibus (GEO) database under the accession codes listed below.
sc-RNA seq: GSE202421 (https://www.ncbi.nlm.nih.gov/geo/query/acc.cgi?acc=GSE202421)
HSC RNA-seq: GSE202463 (https://www.ncbi.nlm.nih.gov/geo/query/acc.cgi?acc=GSE202463)
HSC/MPP HyperTRIBE RNA-seq: GSE202464 (https://www.ncbi.nlm.nih.gov/geo/query/acc.cgi?acc=GSE202464)
Proteomic mass spectrometry data are available via ProteomeXchange with identifier PXD019779

Field-specific reporting

Please select the one below that is the best fit for your research. If you are not sure, read the appropriate sections before making your selection.

☒ Life sciences ☐ Behavioural & social sciences ☐ Ecological, evolutionary & environmental sciences

For a reference copy of the document with all sections, see nature.com/documents/nr-reporting-summary-flat.pdf

Life sciences study design

All studies must disclose on these points even when the disclosure is negative.

Sample size	For the animal studies we analyzed at least 5 animals per group to provide at least 80% power to detect at least 10% difference between mean (with standard deviation equal or less than half of difference between mean) value of control (WT) vs. tested (KO) group at the 0.05 significance level. For other studies, no sample size calculation was performed for these experiments. For in vitro experiments in mouse cells, sequencing, proteomics, and western blot we aimed for a number of at least 3 independent experiments per group to allow for statistical inference using Student's t test (two tailed unless otherwise stated).
Data exclusions	No data were excluded from analysis.
Replication	For each experiments, at least 3 independent biological replicates were performed.
Randomization	We allocated recipient mice into different groups randomly in transplant in vivo experiments. Animals in all experiment groups are sex and age matched. No other randomization was performed in the study.
Blinding	The authors were not blinded. Validation of efficient depletion of SYNCRIP - the gene of interest - must be confirmed prior to experimental assessments.

Reporting for specific materials, systems and methods

We require information from authors about some types of materials, experimental systems and methods used in many studies. Here, indicate whether each material, system or method listed is relevant to your study. If you are not sure if a list item applies to your research, read the appropriate section before selecting a response.

Materials & experimental systems

n/a	Involved in the study	
☐	☒	Antibodies
☐	☒	Eukaryotic cell lines
☒	☐	Palaeontology and archaeology
☐	☒	Animals and other organisms
☒	☐	Human research participants
☒	☐	Clinical data
☒	☐	Dual use research of concern

Methods

n/a	Involved in the study	
☒	☐	ChIP-seq
☐	☒	Flow cytometry
☒	☐	MRI-based neuroimaging

Antibodies

Antibodies used	Target antigen and clone; Vendor; Catalog number; Application (Dilution)<br>Syncrip Ab (anti-hnRNP Q, clone 7A11.2); Millipore Sigma; Cat# MAB11004; Western Blot (1:1000); Immunofluorecence (1:500)

Actin Ab; Sigma Aldrich; Cat# A3854; Western Blot (1:20000)
Gr1-APC RB6-8C5; Thermo Fisher Scientific; Cat# 17-5931-82; Flow cytometry (1:200)
Mac1-PB clone M1/70; Biolegend; Cat# 101224; Flow cytometry (1:200)
cKit- APC-Cy7 clone 2B8; Biolegend; Cat# 105826; Flow cytometry (1:200)
B220-PE Cy7 clone RA3-6B2; BD Biosciences; Cat# 552772; Flow cytometry (1:200)
CD19-PE Cy5 clone eBio1D3 (1D3); Thermo Fisher Scientific; Cat# 15-0193-83; Flow cytometry (1:200)
IgM-APC clone RMM-1; Biolegend; Cat# 406509; Flow cytometry (1:200)
CD3e-PB clone 145-2C11; Thermo Fisher Scientific; Cat# 48-0031-82; Flow cytometry (1:200)
CD4-PE clone GK1.5; BD Biosciences; Cat# 557308; Flow cytometry (1:200)
CD8-FITC clone clone 53-6.7; BD Biosciences; Cat# 553031; Flow cytometry (1:200)
Sca1-PB clone E13-161.7; Biolegend Cat# 122520; Flow cytometry (1:200)
CD150-APC clone TC15-12F12.2; Biolegend; Cat# 115910; Flow cytometry (1:200)
CD48-PE clone HM48-1; BD Biosciences; Cat# 557485; Flow cytometry (1:200)
CD41-PE clone GK1.5; BD Biosciences; Cat# 557308; Flow cytometry (1:200)
Ter119-PE Cy5 clone TER-119; Thermo Fisher Scientific; Cat# 15-5921-82; Flow cytometry (1:200)
CD71-FITC clone R17217; Thermo Fisher Scientific; Cat# 11-0711-81; Flow cytometry (1:200)
CD45.1- PE CF594 clone A20; BD Biosciences; Cat# 562452; Flow cytometry (1:200)
CD45.2-Alexa 700 clone 104; Thermo Fisher Scientific; Cat# 56-0454-82; Flow cytometry (1:200)
CD3e-PE Cy5 clone 145-2C11; Invitrogen; Cat# 15-0031-83; Flow cytometry (1:200)
CD4-PE Cy5 clone GK1.5; Invitrogen; Cat# 15-0041-83; Flow cytometry (1:200)
CD8-PE Cy5 clone 53-6.7; Invitrogen; Cat# 15-0081-83; Flow cytometry (1:200)
Gr1-PE Cy5 clone RB6-8C5; Invitrogen; Cat# 15-5931-82; Flow cytometry (1:200)
B220-Pe Cy5 clone RA3-6B2; Invitrogen; Cat# 15-0452-83; Flow cytometry (1:200)
Numb Ab; Abcam; Cat# Ab4147; Immunofluorescence (1:500)
CDC42 Ab; Abcam; Cat# ab64533; Immunofluorescence (1:500)
Tubulin Ab; Abcam; Cat# ab6160; Immunofluorescence (1:500)
Lamp1 Ab; Abcam; cat# ab208943; Immunofluorescence (1:500)
Chop Ab; Cell Signaling Technology; Cat#2895; Immunofluorescence (1:100)
ATF4 Ab; Cell Signaling Technology; Cat#11815; Immunofluorescence (1:100)
HSP70 Ab; Abcam; Cat#ab181606; Immunofluorescence (1:100)
Alexa Fluor 488 donkey anti-goat; Invitrogen; Cat#A11055; Immunofluorecence (1:500)
Alexa Fluor 568 donkey anti-rabbit; Invitrogen; Cat#A10042; Immunofluorecence (1:500)
Alexa Fluor 647 donkey anti-mouse; Invitrogen; Cat#A31571; Immunofluorecence (1:500)
Alexa Fluor 488 goat anti-rat; Invitrogen; Cat#A11006; Immunofluorecence (1:500)
Alexa Fluor 568 goat anti-rat; Invitrogen; Cat#A11007; Immunofluorecence (1:500)
Alexa Fluor 647 donkey anti-rabbit; Invitrogen; Cat#A31573; Immunofluorecence (1:500)
DRAQ5; Abcam; Cat#ab108410; Immunofluorecence (1:1000)

Validation

All antibodies used were purchased from commercial vendors and have been validated by the vendors. Information on each antibody and their validated application is available at the manufacture's website, we also list the validation for each primary antibody below. We used only for validated applications. In addition, SYNCRIP antibody is confirmed by our knockout mouse cells. SYNCRIP antibody is validated in mice for western blot and immunofluorecence applications according to the data in our manuscript (Figures 1B, 2A, 6A, 6B). We note in the manuscript, this antibody is not specific to only SYNCRIP, and also detects HNRNPR. We detail in the Western blots where HNRNPR is relative to SYNCRIP.

Primary Antibody Validation:
Actin (https://www.sigmaaldrich.com/US/en/product/sigma/a3854): For application in Western Blots. Species reactivity with sheep, carp, feline, chicken, rat, mouse, Hirudo medicinalis, rabbit, canine, pig, human, bovine, guinea pig.
NUMB (https://www.abcam.com/products/primary-antibodies/numb-antibody-ab4147.html): Statement from manufacture lists that their Abpromise guarantee policy covers the use of ab4147 in the following tested applications: ELISA, IHC-P, IP, WB, ICC/IF. With species reactivity to Mouse, Chicken, Human.
CDC42 (https://www.abcam.com/products/primary-antibodies/cdc42-antibody-ab64533.html): Statement from manufacture lists that their Abpromise guarantee policy covers the use of ab64533 in the following tested applications: ICC/IF and WB. With species reactivity to Mouse and Human.
TUBULIN (https://www.abcam.com/products/primary-antibodies/tubulin-antibody-yl12-loading-control-ab6160.html): Statement from manufacture lists that their Abpromise guarantee policy covers the use of ab6160 in the following tested applications: WB, Flow Cytometry, ICC/IF, IHC-P.
LAMP1 (https://www.abcam.com/products/primary-antibodies/lamp1-antibody-epr21026-ab208943.html): Statement from manufacture lists that their Abpromise guarantee policy covers the use of ab208943 in the following tested applications: WB, IHC-P, IHC-Fr, IP, ICC/IF, Flow Cytometry. With species reactivity to mouse.
CHOP (https://www.cellsignal.com/products/primary-antibodies/chop-l63f7-mouse-mab/2895?_=1679433030183&Ntt=2895&tahead=true): Manufacture's antibody guarantee policy ensures that antibodies are validated in house for the certain research applications. CHOP (L63F7) Mouse mAb #2895 is validated for the following applications: WB, IP, IF, Flow Cytometry, Chromatin IP. With species reactivity to human, mouse and rabbit.
ATF4 (https://www.cellsignal.com/products/primary-antibodies/atf-4-d4b8-rabbit-mab/11815): Manufacture's antibody guarantee policy ensures that antibodies are validated in house for the certain research applications. ATF-4 (D4B8) Rabbit mAb #11815 is validated for the following applications: WB, IP, IF, Chromatin IP, Chromatin IP-seq, Cut & Run. With Species reactivity to human, mouse and rabbit.
HSP70 (https://www.abcam.com/products/primary-antibodies/hsp70-antibody-epr16892-ab181606.html): Statement from manufacture lists that their Abpromise guarantee policy covers the use of ab181606 in the following tested applications: Flow Cytometry, IHC-P, WB, ICC/IF. With species reactivity to mouse, rat, and human.
DRAQ5 (https://www.abcam.com/products/reagents/draq5-ab108410.html): Statement from manufacture lists that their Abpromise guarantee policy covers the use of ab108410 in the following tested applications: FM, Flow Cytometry, ICC/IF. For use as a cell-permeant DNA intercalating probe in fixed or non-fixed/ live cells.
Gr1-APC RB6-8C5 (https://www.thermofisher.com/antibody/product/Ly-6G-Ly-6C-Antibody-clone-RB6-8C5-Monoclonal/17-5931-82): Application Flow Cytometry, species reactivity Dog, Human, Mouse.

Mac1-PB clone M1/70 (https://www.biolegend.com/en-us/products/pacific-blue-anti-mouse-human-cd11b-antibody-3863): Application Flow Cytometry; Species Reactivity: Mouse, Human, Cynomolgus, Rhesus
cKit- APC-Cy7 clone 2B8 (https://www.biolegend.com/en-us/products/apc-cyanine7-anti-mouse-cd117-c-kit-antibody-5905): Application Flow Cytometry; Species Reactivity: Mouse
B220-PE Cy7 clone RA3-6B2 (https://www.bdbiosciences.com/en-us/products/reagents/flow-cytometry-reagents/research-reagents/single-color-antibodies-ruo/pe-cy-7-rat-anti-mouse-cd45r-b220.552772): Application Flow Cytometry; Species Reactivity: Mouse
CD19-PE Cy5 clone eBio1D3 (1D3) (https://www.thermofisher.com/antibody/product/CD19-Antibody-clone-eBio1D3-1D3-Monoclonal/15-0193-82): Applications Flow Cytometry; Species Reactivity: Human, Mouse
IgM-APC clone RMM-1 (https://www.biolegend.com/en-us/products/apc-anti-mouse-igm-2335): Application Flow Cytometry; Species Reactivity Mouse.
CD3e-PB clone 145-2C11 (https://www.thermofisher.com/antibody/product/CD3e-Antibody-clone-145-2C11-Monoclonal/48-0031-82): Application Flow Cytometry; Species Reactivity Mouse.
CD4-PE clone GK1.5 (https://www.bdbiosciences.com/en-us/products/reagents/flow-cytometry-reagents/research-reagents/single-color-antibodies-ruo/pe-rat-anti-mouse-cd4.557308): Application Flow Cytometry; Species Reactivity Mouse.
CD8-FITC clone clone 53-6.7 (https://www.bdbiosciences.com/en-us/products/reagents/flow-cytometry-reagents/research-reagents/single-color-antibodies-ruo/fitc-rat-anti-mouse-cd8a.553031): Application Flow Cytometry; Species Reactivity Mouse.
Sca1-PB clone E13-161.7 (https://www.biolegend.com/en-us/products/pacific-blue-anti-mouse-ly-6a-e-sca-1-antibody-3901): Applications flow cytometry; species reactivity mouse.
CD150-APC clone TC15-12F12.2 (https://www.biolegend.com/en-us/products/apc-anti-mouse-cd150-slam-antibody-2894): Applications flow cytometry; species reactivity mouse.
CD48-PE clone HM48-1 (https://www.bdbiosciences.com/en-us/products/reagents/flow-cytometry-reagents/research-reagents/single-color-antibodies-ruo/pe-hamster-anti-mouse-cd48.557485): Application flow cytometry; species reactivity mouse.
CD41-PE clone GK1.5 (https://www.bdbiosciences.com/en-us/products/reagents/flow-cytometry-reagents/research-reagents/single-color-antibodies-ruo/pe-rat-anti-mouse-cd41.558040): Application Flow Cytometry; species reactivity mouse.
Ter119-PE Cy5 clone TER-119 (https://www.thermofisher.com/antibody/product/TER-119-Antibody-clone-TER-119-Monoclonal/15-5921-82): Application flow cytometry; species reactivity Fish, Human, Mouse.
CD71-FITC clone R17217 (https://www.thermofisher.com/antibody/product/CD71-Transferrin-Receptor-Antibody-clone-R17217-RI7-217-1-4-Monoclonal/11-0711-81): Application Flow Cytometry; Species Reactivity Mouse.
CD45.1- PE CF594 clone A20 (https://www.bdbiosciences.com/en-us/products/reagents/flow-cytometry-reagents/research-reagents/single-color-antibodies-ruo/pe-cf594-mouse-anti-mouse-cd45-1.562452): Application flow cytometry; species reactivity mouse.
CD45.2-Alexa 700 clone 104 (https://www.thermofisher.com/antibody/product/CD45-2-Antibody-clone-104-Monoclonal/56-0454-82): Application flow cytometry; Species reactivity Mouse
CD3e-PE Cy5 clone 145-2C11 (https://www.thermofisher.com/antibody/product/CD3e-Antibody-clone-145-2C11-Monoclonal/15-0031-83): Application flow cytometry; species reactivity mouse.
CD4-PE Cy5 clone GK1.5 (https://www.thermofisher.com/antibody/product/CD4-Antibody-clone-GK1-5-Monoclonal/15-0041-83): Application flow cytometry; species reactivity mouse.
CD8-PE Cy5 clone 53-6.7 (https://www.thermofisher.com/antibody/product/CD8a-Antibody-clone-53-6-7-Monoclonal/15-0081-83): Application flow cytometry; species reactivity mouse.
Gr1-PE Cy5 clone RB6-8C5 (https://www.thermofisher.com/antibody/product/Ly-6G-Ly-6C-Antibody-clone-RB6-8C5-Monoclonal/15-5931-82): Application flow cytometry, species reactivity mouse.
CD43 FITC clone S7 (https://www.bdbiosciences.com/en-us/products/reagents/flow-cytometry-reagents/research-reagents/single-color-antibodies-ruo/fitc-rat-anti-mouse-cd43.553270): Application flow cytometry; species reactivity mouse.
B220-Pe Cy5 clone RA3-6B2 (https://www.thermofisher.com/antibody/product/CD45R-B220-Antibody-clone-RA3-6B2-Monoclonal/15-0452-83): Application flow cytometry; species reactivity: human,mouse.

Eukaryotic cell lines

Policy information about cell lines

Cell line source(s)
All cells used for experiments were from primary sources and not cell lines. HEK 293T cells were used to generate retrovirus.

Authentication
All cells used for experiments were from primary sources and not cell lines. HEK 293T cells were not authenticated.

Mycoplasma contamination
Cultured cells were routinely tested and confirmed negative for mycoplasma in house by using a Mycoplasma Test from Lonza Biosciences (#LT07-218) according to manufacturer's instructions.

Commonly misidentified lines (See ICLAC register)
No commonly misidentified cell lines were used in this study.

Animals and other organisms

Policy information about studies involving animals; ARRIVE guidelines recommended for reporting animal research

Laboratory animals
MSKCC animal facility is maintained at temperatures between 64 -78° F, with humidity of the animal room ranging between 30-70%. 6-8 week old female B6.SJL-Ptprc <a>/Boy (Taconic Bioschences, USA) mice were used as recipients for in vivo transplant experiments (lethally irradiated as described in methods). Mx-1 Cre 8-12 week old male mice were used to cross with Syncrip f/f strain to create Syncrip cKO strain.

Wild animals
No wild animals were used in this study.

Field-collected samples
No field-collection was used in this study.

Ethics oversight	All animal studies were performed on the animal protocol, #11-10-025, approved by the Institutional Animal Care and Use Committee (IACUC) at Memorial Sloan Kettering Cancer Center.

Note that full information on the approval of the study protocol must also be provided in the manuscript.

Flow Cytometry

Plots

Confirm that:

☒ The axis labels state the marker and fluorochrome used (e.g. CD4-FITC).

☒ The axis scales are clearly visible. Include numbers along axes only for bottom left plot of group (a 'group' is an analysis of identical markers).

☒ All plots are contour plots with outliers or pseudocolor plots.

☒ A numerical value for number of cells or percentage (with statistics) is provided.

Methodology

Sample preparation	Blood or bone marrow samples were lysed of red blood cells, and were subsequently stained with the surface marker antibody, dye or probe according to each appropriate experiment. For surface marker staining, cells were incubated for 30 minutes and washed with PBS then resuspended in RPMI 2% FBS for flow cytometry. All other probes were incubated according to previous publications, as described in Methods.
Instrument	BD FACS Fortessa equipment for Flow cytometry analysis and BD FACS Solo-Aria II for sorting.
Software	FlowJo version 10.6.2
Cell population abundance	For flow analysis, samples were run to acquire at least 10,000 events in gated cell population. Analyzed populations were back-gated to confirm purity.<br>For RNA-sequencing analysis of sorted populations, gene expression profiles of sorted cells were used to validate cell identities by geneset enrichment analysis (GSEA).
Gating strategy	Cells were gated for live cells (FSC-A, SSC-A), then for singlets (single cells) with FSC-H and FSC-W and SSC-H and SSC-W. Cells were then gated according to analysis in specific experiment, described in figure legends. Comparisons between control and experimental cells were done on the same gating strategies.

☒ Tick this box to confirm that a figure exemplifying the gating strategy is provided in the Supplementary Information.
